# primetv: a viewer for reconciled trees

**DOI:** 10.1186/1471-2105-8-148

**Published:** 2007-05-07

**Authors:** Bengt Sennblad, Eva Schreil, Ann-Charlotte Berglund Sonnhammer, Jens Lagergren, Lars Arvestad

**Affiliations:** 1 Stockholm Bioinformatics Center and Department of Biochemistry and Biophysics, Stockholm University, SE-106 91, Stockholm, Sweden; 2 Stockholm Bioinformatics Center and School of Computer Science and Communication, KTH, SE-100 44, Stockholm, Sweden; 3 Now at Linnaeus Centre for Bioinformatics and Uppsala Multidisciplinary Center for Advanced Computational Science, Uppsala University, BMC, Box 598, SE-751 24 Uppsala, Sweden

## Abstract

**Background:**

Evolutionary processes, such as gene family evolution or parasite-host co-speciation, can often be viewed as a tree evolving inside another tree. Relating two given trees under such a constraint is known as reconciling them. Adequate software tools for generating illustrations of tree reconciliations are instrumental for presenting and communicating results and ideas regarding these phenomena. Available visualization tools have been limited to illustrations of the most parsimonious reconciliation. However, there exists a plethora of biologically relevant non-parsimonious reconciliations. Illustrations of these *general *reconciliations may not be achieved without manual editing.

**Results:**

We have developed a new reconciliation viewer, primetv. It is a simple and compact visualization program that is the first automatic tool for illustrating *general *tree reconciliations. It reads reconciled trees in an extended Newick format and outputs them as tree-within-tree illustrations in a range of graphic formats. Output attributes, such as colors and layout, can easily be adjusted by the user. To enhance the construction of input to primetv, two helper programs, readReconciliation and reconcile, accompany primetv. Detailed examples of all programs' usage are provided in the text. For the casual user a web-service provides a simple user interface to all programs.

**Conclusion:**

With primetv, the first visualization tool for *general *reconciliations, illustrations of trees-within-trees are easy to produce. Because it clarifies and accentuates an underlying structure in a reconciled tree, e.g., the impact of a species tree on a gene-family phylogeny, it will enhance scientific presentations as well as pedagogic illustrations in an educational setting. primetv is available at , both as a standalone command-line tool and as a web service. The software is distributed under the GNU General Public License.

## Background

Illustrating evolution using phylogenetic trees is a natural way of depicting evolutionary processes. Indeed, already Darwin drew trees and there exist many tools for automatically producing graphical representations of phylogenies. Some phenomena are, however, better viewed as trees-within-trees. For example, gene family evolution should be put in the context of species evolution, parasite evolution is best understood in relation to a host tree, and a speciation process that is affected by geological processes, such as continental breakup and movement, is often viewed as a species tree that evolves within an area tree. The relation between the two trees is called a *reconciliation *[1]. More precise, a reconciliation describes how a *guest tree*, *G *(e.g., a gene tree in the case of gene evolution), evolves *inside *a *host tree*, *S *(e.g., a species tree), and allows the prediction and dating of evolutionary events (e.g., gene duplications or speciations) corresponding to vertices of *G *with respect to *S*. A special case is the *most parsimonious reconciliation*, *MPR*, i.e., the reconciliation that minimizes the number of evolutionary events. In order to avoid confusion, we will here use the term *general reconciliation *to indicate the set of all possible reconciliations (i.e., *not *restricted to *MPR*s).

Illustrations of these phenomena are common in the literature today, but, apart from *MPR*s, graphic representations of general reconciliations must be created by hand. Early support for viewing *MPR*swas available in COMPONENT [2], GeneTree [3] and TreeMap [4], featuring a nested view of co-evolving trees as well as an opportunity to look at them side-by-side. The Mesquite [5] coalescence package also provides automatic calculation and viewing of *MPR*s. The tree viewer ATV [6] has support for displaying vertices corresponding to gene duplications in a different color, thus implicitly defining the reconciliation, but the relation to the species tree is not directly visualized. The ATV interface has been inherited and improved in Notung [7], which is a program for e.g. gene family analysis, including methods for tree reconstruction, *MPR*sand gene-tree rooting. The RAP system [8] visualizes *MPR*s similar to ATV, but also  supports interactions with databases through a graphical  query language for gene tree features, thereby helping the  user find gene families with a given evolutionary pattern. DupLoss [9] displays *MPR*sbut is an exploratory tool for algorithmic research rather than a general reconciliation viewer and cannot, for example, read input with arbitrary leaf names. Another approach to identify duplications, that implicitly relies on  *MPR*s, is taken by TreeSimplifier [10], which shows guest  trees on a hyperbolic surface and supports collapsing  subtrees that are isomorphic to the host tree. Finally, we  mention TreeDyn [11], a powerful tree editor that, while not  directly handling reconciliations, allows visualization of  relations between leaves in host trees and guest trees. We present primetv (*PrIME *Tree Viewer, PrIME is Probabilistic Inference of Models of Evolution), to our knowledge the first computer program that produce tree-within-tree illustrations of general reconciliations, i.e., not restricted to *MPR*s. For convenience, a primetv web service is also available where a user can create illustrations without installing any software.

## Implementation

Input to primetv is a host tree, *S *(typically a species tree), and a reconciled tree, (*G*, *γ*), where the guest tree, *G*, typically is a gene tree and *γ *is a reconciliation between *G *and *S*. Formally, the reconciliation is a mapping between vertices in *G *and the edges in *S *on which they evolve. A *reconciled tree *is a guest tree given together with a reconciliation.

**Figure 1:**
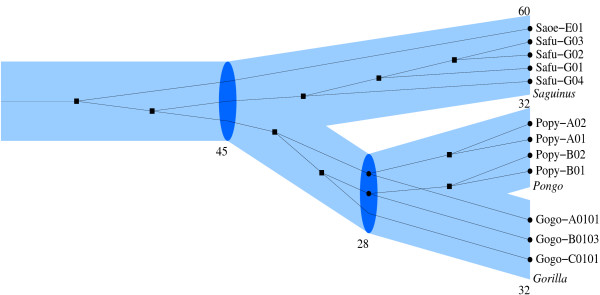
**An example reconciliation**. primetv-generated illustration of the reconciled tree showing the evolution of the gene family Major Histocompatibility Complex class I in Gorilla, Orangutan, and Tamarin. The species tree is shown in blue and the gene tree in black. Gene tree vertices corresponding to speciations are indicated by circles and are placed inside species tree vertices, while duplications are indicated by squares. Species names are given in italics and gene names in standard format. The numbers below species tree vertices indicate the time intervals corresponding to the incoming edge, the length of which is scaled according to this number.

**Figure 2:**
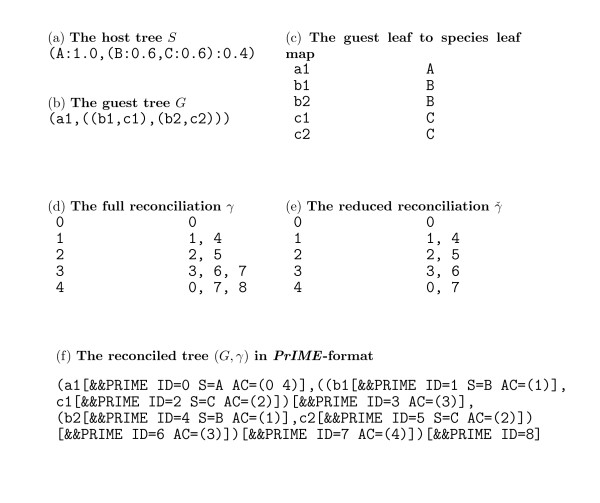
**Input formats of primetv**. **(a) **The host tree, *S*, and **(b) **the guest tree, *G*, in Newick format. **(c) **The leaf map between the host tree in (a) and the guest tree in (b) in tabular form. In each row, the left column has a guest tree leaf name and the right column has the associated host tree leaf name. **(d, e, f) **The reconciliation *γ *can be input to primetv in three different formats: **(d) **The *full *reconciliation, *γ*, in tabular format. For a host tree node *x *(the left column), *γ*(*x*) (the right column of the same row) comprise all guest tree vertices whose incoming edges appear on the incoming edge of *x *in the reconciled tree, cf. *γ*(4) as indicated in Figure 3(a). Notice that a guest vertex can map to several host vertices. **(e) **The *reduced *reconciliation, , in tabular format. This is achieved from a full reconciliation, *γ*, by, for each host tree vertex *x*, removing from *γ*(*x*) all guest vertices that are ancestral to other vertices in *γ*(*x*), e.g., 8 in *γ*(4). **(f) **The reconciled tree (*G*, *γ*) in *PrIME *format. This is a Newick tree with *PrIME *tags added to tree vertices; a sequence of *PrIME *tags are always given within brackets and are preceded by the tag specifier &&PRIME. For a guest vertex, *v*, the tag ID indicates a unique number identifying *v*. If no ID-tag is given, e.g., in Newick format, identity numbers are assigned automatically. The tag AC indicates the ID-numbers of the host tree vertices that *v *maps to in the reduced reconciliation ; these host tree vertices should always form a path in the host tree. Additionally, for a leaf *l*, the tag S indicate the label of the host tree leaf that *l *maps to. When reading host trees primetv will assume that numbers after colons (:) represent (ultrametric) edge times rather than edge lengths. This can be made explicit by using the tag ET in the *PrIME *format.

The output of primetv is an illustration of *S*, *G *and *γ *that has *G *nested in *S *(see Figure 1 for an example): The host tree *S *is drawn using wide edges and ellipses for vertices. The guest tree is drawn using narrow edges and circles or squares for the vertices: Vertices in *G *that *γ *associates with vertices in *S*, i.e., speciations in the case of a gene family, are marked by circles and placed within the corresponding ellipses of *S*. The remaining vertices in *G*, i.e., duplications in gene family evolution, are marked by squares and are evenly distributed over edges in *S*. There is a number of options affecting colors and format of the output; these are further described in the *Results and Discussion *section.

primetv is written to work closely with output from *PrIME *(PRobabilistic Integrated Models of Evolution), a line of tools we are writing for performing probabilistic tree reconciliations [12,13]. Thus, reconciled trees from these computer programs can be used as input directly. The *PrIME *reconciled tree format is documented in detail at the primetv web site. In brief, it is based on the Newick [14] tree format. A reconciled guest tree may have additional vertex markups, of the type used in the Nexus [15] and NHX [16] formats, that indicates the host tree vertex to which it is reconciled, see example in Figure 2(f). A user can take any phylogenetic tree, add markup following the *PrIME *format, and view manually constructed reconciliations. Since such an exercise is quite tedious and error prone, we provide two helper programs to simplify this task.

The first, readReconciliation, is a translator that takes a guest tree, a host tree, and a separate reconciliation description and returns a reconciled tree in *PrIME*-format. The input guest and host trees are in Newick or *PrIME*-format, and the input reconciliation is in tabular form, see Figure 2(d) and 2(e) and the *Results and Discussion *section. The output is in *PrIME*-format and can be directly fed into primetv. The second helper program, reconcile, is an implementation of Zhang's algorithm [17] for most parsimonious tree reconciliation. For simplicity of implementation, a quadratic time version of the least-common-ancestor subroutine was used. Its input is in the Newick or *PrIME *format and the output is either a reconciled tree in *PrIME*-format that can be directly fed to primetv, or a reconciliation in tabular format that can provide a starting point for a user's adaptation to non-*MPR *reconciliations.

The programs are all implemented using C++. A simple graphical user interface is provided for the web-service. The GNU plotutils [18] package was used to generate the graphics. It has the advantage that we can produce output in a number of practical formats. Besides the popular bitmap-based image formats such as GIF and PNG, there is EPS, SVG, and FIG for vector-based illustrations that also can be manipulated and improved manually with programs such as Adobe Illustrator (Adobe Systems Inc.), Inkscape [19] and XFig [20].

## Results and Discussion

To illustrate the competence of primetv we give two examples of its application, illustrating different inputs to primetv. These two examples also illustrate the formatting options of primetv.

The first example is a test case from the major histocompatibility complex class I, *MHCI*, in primates, analyzed using *PrIME *in [9,10]. The species tree consists of three primates, its branch times are taken from [21], and the gene tree is from [22]. The reconciliation is estimated with *PrIME *and the reconciled tree from *PrIME *is used as input to primetv. The output from primetv is presented in Figure 1.

The relation between the gene tree and the species tree is clear when using primetv: Identifying the gene tree's speciation vertices and their corresponding vertices in the species tree is straightforward. Gene losses are also apparent from missing gene lineages at speciations in the species tree. Another advantage with this type of illustration is that deciding which species a gene belongs to is immediate; you do not need to rely on a prefix or suffix notation in the gene names. Notice that the reconciliation in Figure 1 is not the *MPR*: the second gene tree vertex from the left in Figure 1 would in the *MPR *be denoted as a speciation. However, as described in [12,13], if auxiliary evidence, i.e., *MHCI *representatives from the human genome, is included it becomes evident, also with the most parsimonious reconciliation, that this vertex indeed represent a duplication. The ability to output non-*MPR *illustrations is currently unique to primetv.

**Figure 3:**
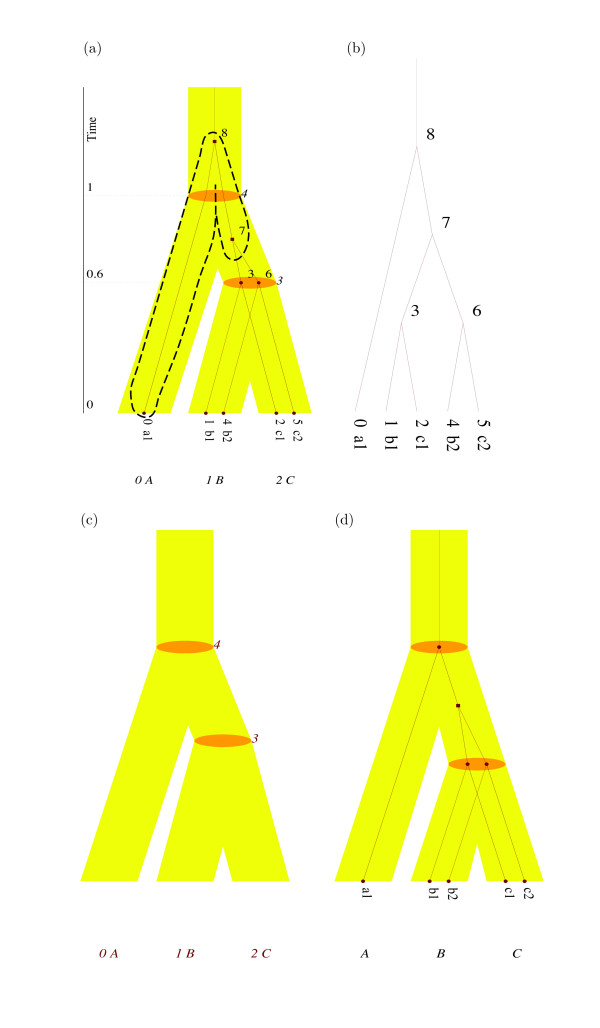
**primetv output versatility**. primetv output illustrations associated with the data given in Figure 2. **(a) **The reconciled tree (*G*, *γ*), with *γ*(4) indicated with a dashed line. **(b, c) **The guest tree- and host tree-parts, respectively, of the reconciled tree (*G*, *γ*) in Figure 3(a). **(d) **The most parsimonious reconciled tree between the host tree and the guest tree in Figure 2. The host tree is given in yellow and orange and the guest tree is given in dark-red. Numbers by vertices indicate the unique vertex identity labels assigned by *PrIME *. In **(a)**, divergence times for host tree vertices are given on a time scale to the left. Dashed line in **(a) **is added, posterior to primetv output, using Adobe Illustrator (Adobe Systems Inc.).

The second example illustrates how to manually construct input to primetv using readReconciliation or reconcile. Assume that we want to reconcile the guest tree *G *to the host tree *S*, Figure 3(b) and 3(c) respectively, to achieve the reconciled tree in Figure 3(a). The input to readReconciliation comprises *S *and *G *in Newick- (Figure 2(a) and 2(b), respectively) or *PrIME*-format, and a reconciliation in tabular format as described below. The reconciliation associates vertices in *S *with vertices in *G *using their unique identity number. The *PrIME *programs, including primetv, automatically assign such identity numbers in a consistent way, and primetv can be used to view the assigned identity numbers, e.g., Figure 3(b) and 3(c) shows automatically assigned identity numbers on *G *and *S*, respectively. However, as a convenience user-defined identity numbers (that override the automatic identity numbers) can be assigned using the tag ID in the tree file, as exemplified in Figure 2(f).

The tabular format for the reconciliation is given as a two column table where, for each row, the left column holds the identity number of a host tree vertex and the right column holds the identity numbers of the guest tree vertices reconciled to this host tree vertex. Depending on how the guest tree vertices are given, there are two 'flavors' of the tabular format. In the *full *reconciliation, denoted *γ*, the right column comprise the guest tree vertices whose incoming edge appear on the incoming edge of the host tree vertex. An example is given in Figure 2(d) and row 4 in this table, i.e., *γ*(4) is graphically indicated in Figure 3(a). Notice that the guest vertices mapped to a host tree vertex in *γ *form subtrees of *G*. The *reduced *reconciliation, denoted , comprises for each host vertex the leaves of these subtrees, cf. Figure 2(e). Reduced reconciliations are generic in *PrIME*, but full reconciliations may be more intuitive for users. The result of readReconciliation run with *S*, *G *and either *γ *or  as arguments is the reconciled tree (*G*, *γ*) shown in Figure 2(f). Here the tag AC for a guest tree vertex *v *comprise the host vertices to which *v *is mapped in the reduced reconciliation (i.e., the inverse of ). The primetv output for this reconciled tree is given in Figure 3(a).

The program reconcile produces most parsimonious reconciliations, *MPR*s, of trees. It takes as input a host tree and a guest tree in Newick format and a leaf-map in tabular format that maps the name of a guest tree leaf to the name of the corresponding host tree leaf, see example in Figure 2(c). The output is either the most parsimonious reconciliation, γ*, in tabular format, or the reconciled tree (*G*, γ*) in *PrIME *format. The output reconciliation in tabular form may be used as a starting point for the construction of user-defined reconciliations. The reconciled tree output can be fed directly to primetv; the most parsimonious reconciled tree for the host tree and guest tree in Figure 2 is shown in Figure 3(d).

As a further convenience, the programs can be accessed using the web interface, . There, guest and host trees in *PrIME *format are put into text boxes. The user can then choose between creating an *MPR *or giving a reconciliation on a tabular or *PrIME *format; if the *MPR *alternative is chosen, an additional leaf map (which for easy cases is deduced automatically) is needed as input to reconcile. In either case a primetv-generated illustration is presented along with options for layout and color changes. The computed reconciliation is also given in a text box to allow for modifications and immediate updates of the illustration.

Figures 1 and 3 also illustrate the customization options available in primetv. The layout can be altered in various ways: The tree in Figure 1 is drawn horizontally, ladderized right and with the subtrees positioned asymmetrically, while the tree in Figure 3(a) is drawn vertically, ladderized right and with subtrees distributed symmetrically. In these figures, the host tree edges are scaled in relation to their edge times; This scaling, however, can be removed as in Figure 3(d). It is also possible to remove either the guest tree, as in Figure 3(c), or the host tree, as in Figure 3(b). For this last option no host tree needs to be given, thus primetv can be used to illustrate any tree, i.e., not only reconciled trees. Colors of guest and host tree vertices and edges are highly customizable and examples are shown in Figures 1 and 3. Some annotation can also be customized. The time annotation of the host tree can be shown as divergence times on a time axis, as in Figure 3(a), or as the time intervals associated with host tree edges, as in Figure 1. As shown in Figure 3(b) and 3(c), vertex annotation with identity numbers assigned by *PrIME *can be shown. Further options not shown include scaling of fonts and change of page size or bitmap size. The result can be shown on an X11-window or saved to file in a number of formats, see section *Implementation*.

## Conclusion

Our tool primetv is a simple and compact computer program dedicated to visualizing reconciled trees. It is the first tool to visualize *general *reconciliations and not only *MPR*s. primetv visualizes reconciled trees as trees within trees. This type of illustration clarifies and accentuates the underlying structure in a reconciled tree, for example the impact of a species tree on a gene family phylogeny or how parasite evolution has followed its host species. Many visualization attributes, such as colors and layout parameters, can be adjusted according to the user's needs, thus making it possible to adapt the style of the illustration to the style of a presentation. The range of output formats make adaptation easier as well.

These attributes makes primetv a powerful tool for illustrations of gene family histories, orthology analysis, biogeography and co-speciation studies, especially since primetv allows for alternative reconciliations. primetv has formats suitable for manuscript preparation, immediate inspection, web applications, and presentations. The clarity and intuitiveness of trees-within-trees illustrations is also ideal for pedagogic illustrations of the above-mentioned processes in an educational setting.

Two programs distributed jointly with primetv simplify input adaption by removing the need to create input by hand. The primary user interface of primetv is the command line, hence it is easy to include it into scripts allowing automatic visualizations in large data sets. For the casual user, the web interface provides a simple GUI and also avoids the need for installing software.

## Availability and requirements

**Project name: **primetv

**Project home page: **

Release 1.5.3 of primetv is also distributed with this manuscript [see Additional file 1].

**Operating systems: **primetv has been tested on Linux systems (Red Hat 9 and Fedora Core 3), Mac OS X (10.3.9 and 10.4.5) and Microsoft Windows (cygwin 1.5.18-1).

**Programming languages: **The software is a command-line tool written in C++. We have compiled primetv using the GNU C and C++ compilers,  both versions 3.4, 4.0.01 (Mac OS X), and 4.1 (Linux). 

The casual user is recommended to use our web server which, besides being platform independent, provides an easy-to-use user interface.

**Other requirements: **The graphics interface of primetv makes use of the GNU plotutils [18] package. There are a couple of adjustments to plotutils needed to compile on Linux using a modern C++ compiler. The patched source are available on our website, where we have also put executable binaries. For Mac OS X and Microsoft Windows, patched versions of plotutils are available, e.g., through MacPorts and Cygwin [23], respectively.

**License: **The software is distributed under the GNU General Public License.

## Authors' contributions

This project was conceived and supervised by BS, ACBS, JL, and LA. ES wrote most of the software. BS and LA contributed code and wrote this article. LA implemented the web interface.

## Supplementary Material

Additional File 1 Relase 1.5.3 of primetv. The source code distribution of primetv. Click here for file
